# Species diversity rises exponentially with the number of available resources in a multi-trait competition model

**DOI:** 10.1098/rspb.2018.1273

**Published:** 2018-08-29

**Authors:** Andres Laan, Gonzalo G. de Polavieja

**Affiliations:** Champalimaud Research, Champalimaud Center for the Unknown, Lisbon, Portugal

**Keywords:** coexistence, neutral theory, multi-trait competition

## Abstract

Theoretical studies of ecosystem models have generally concluded that large numbers of species will not stably coexist if the species are all competing for the same limited set of resources. Here, we describe a simple multi-trait model of competition where the presence of *N* resources will lead to the stable coexistence of up to 2^*N*^ species. Our model also predicts that the long-term dynamics of the population will lie on a neutral attractor hyperplane. When the population shifts within the hyperplane, its dynamics will behave neutrally, while shifts which occur perpendicular to the hyperplane will be subject to restoring forces. This provides a potential explanation of why complex ecosystems might exhibit both niche-like and neutral responses to perturbations. Like the neutral theory of biodiversity, our model generates good fits to species abundance distributions in several datasets but does so without needing to evoke inter-generational stochastic effects, continuous species creation or immigration dynamics. Additionally, our model is able to explain species abundance correlations between independent but similar ecosystems separated by more than 1400 km inside the Amazonian forests.

## 1. Introduction

Understanding species coexistence has been a long-standing problem in ecological research. Early models of species competition struggled to explain how large numbers of species could stably coexist. MacArthur and Levins showed that, in pure resource competition models, the number of coexisting species will generally not exceed the number of limiting resources in the environment [1]. Likewise, in 1970 Robert May considered more complex competition models, where species could also have direct effects on each other's dynamics, and found that stable coexistence would not occur unless species become increasingly decoupled from one another as species diversity increased [2] (decoupled species populations change over time as if they were non-interacting). The theoretical difficulties in explaining coexistence stand in stark contrast to what we observe in nature. Even in apparently featureless environments like the surface water of the ocean, hundreds of plankton species are known to stably coexist [3]. The same conclusion emerges from studies of more complex systems like tropical forests, where more than 200 different species of trees can be found in a single hectare-sized plot [4].

Over the past decade, models have started to emerge which are able to explain coexistence in complex ecosystems if species interactions are constrained to have a certain structure. For example, Posfai *et al.* recently described a chemostat model in which constraint structure imposed by cellular trade-offs facilitated coexistence of a large number of species even in homogeneous and resource-poor environments [5]. A very different model of competition for growth space in trees also leads to stable coexistence of species [6,7]. Here, coexistence originates from the model's zero-sum intransitive competition dynamics and it requires higher order interactions to be robustly stable. Despite recent progress, the problem of large-scale species coexistence has not yet been solved in general. Both the chemostat model and the zero-sum growth space competition model rely on rather specific assumptions about underlying competition dynamics. These dynamics are unlikely to be equally applicable to all known diverse ecosystems and some empirical work indicates that they might not capture some of the specific systems they were designed to model [8]. When the assumptions of the models are violated, species diversity may rapidly collapse (see [5], electronic supplementary material and [7]). Therefore, research is still needed on whether other types of competition models might also lead to stable coexistence of diverse communities.

Here, we study a multi-trait game theory competition model based on a new combinatorial version of the hawk-dove game [9]. We find that when limited by *N* resources, our model will lead to a stable coexistence of up to 2^*N*^ species. The coexisting species will persist indefinitely in a hybrid state where they are simultaneously capable of exhibiting both niche and neutral dynamics, depending on how the population is perturbed. Like the many other models which contain neutral dynamics, our model generates good fits to species abundance distributions in several empirical datasets, though our model does not require modelling dispersal, immigration or ongoing speciation in order to achieve this result. We also explore the implications of our model for species removal experiments and we prove that the invasibility criterion often used to experimentally study coexistence [10] is a sufficient rather than a necessary feature of stable coexistence models. Finally, we show that our model correctly predicts the approximate magnitude of species abundance correlations between similar but dynamically independent ecosystems.

## 2. Methods

### (a) Formulation of the model

Our model took inspiration from the hawk-dove game studied in evolutionary game theory [9,11]. The hawk-dove game is characterized by two strategies: the hawk strategy and the dove strategy. Hawks and doves mix in the population randomly and compete against one another. When two hawks meet, both suffer a fitness loss of (*V* − *C*)/2 (where *V* is the value of the resource they are competing for and *C* is cost of mutual competition, also *C* > *V* > 0). On the other hand, when a hawk meets a dove the dove surrenders and the hawk increases its fitness by *V* while a dove gets nothing. When two doves meet they split the resource and each gets *V*/2 increase in fitness. The game is at equilibrium if the frequency of hawks in the population is equal to *p*_*H*_ = *V*/*C* (see the electronic supplementary material, appendix S1.1 for an extended discussion of a hawk-dove game and its underlying assumptions).

In our extension, each species participates in *N* hawk-dove micro games simultaneously. Each micro game can be thought as being a competition for a different kind of essential resource. Micro game 1 might correspond to competition for light, micro game 2 for competition for nitrogen and so on. Each micro game has its corresponding parameters. For micro game *i*, we denote these with values *V*_*i*_ and *C*_*i*_. Alternatively, the micro games might be thought of as representing competition over niche aspects in a multi-dimensional niche space [12].

Each species must choose a micro strategy (either play hawk or play dove) for each of the *N* micro games. For the case of four micro games, for example, a macro strategy for an individual specifies how to play in the four games. An example macro strategy may be *H*_1_ *D*_2_ *D*_3_ *H*_4_, which specifies to play hawk in game 1, dove in game 2, dove in game 3 and hawk in game 4. Furthermore, we postulate that if two individuals have exactly the same macro strategy then they belong to the same species. The total pay-off of a macro strategy is determined by the sum of pay-offs gathered in all of the *N* micro games.

For *N* micro games there is a total of 2^*N*^ different macro strategies. The state of the population is then characterized by 2^*N*^ probability values *π*_*j*_, where *π*_*j*_ is the probability of encountering macro strategy *j*. The population is at equilibrium when every strategy has the same expected fitness. As all *N* micro games are independent from one another in terms of pay-offs, then the population must be in equilibrium with respect to each of the *N* games. Therefore, the probability of encountering the hawk strategy in game *k* (*p*_*H*_*k*__) must be equal to *p*_*H*_*k*__ = *V*_*k*_/*C*_*k*_ (see the electronic supplementary material, appendix S1.2 for an extended derivation). We can write that condition in terms of the probabilities of the composite strategies as 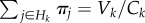, where *j* ∈ *H*_*k*_ stands for all the macro strategies *j* which involve playing hawk in the micro game *k*. We get one such equation for each of the *N* games and another equation which requires that all the probabilities sum to 1. Therefore, we end up with *N* + 1 equations for 2^*N*^ variables and the final solution is under-determined. In effect, the mathematical structure of the solution is constrained to lie somewhere inside a 2^*N*^ − *N* − 1 dimensional hyperplane. Within that hyperplane, all solutions are equally good and therefore there is plenty of room for neutral drift between the strategies.

### (b) A description of empirical datasets

In order to test our new model, we numerically computed the species abundance distributions that the model predicts in equilibrium (see the electronic supplementary material, appendix S1.3) and we compared the simulation results against empirical species abundance distributions. In particular, we use data from the Barro Colorado tree community [13] and measurements of Mediterranean plankton species richness [14]. The Barro Colorado dataset relies on a comprehensive census of 50 hectare-sized sites of tropical forest in Panama. A comprehensive census identified all trees with a diameter larger than 10 cm at breast height (1.3 m). The resulting dataset of species abundances is publicly available and was downloaded from [13]. The plankton dataset was obtained by doing a count of all the species visible after fixation with an inverted Utermohl microscope. The total volume of water examined was 5 l. The water samples originated near the coast of Spain with exact sampling locations described in [14]. The data of species counts was provided in table 2 of [14].

### (c) Modelling species abundance correlations

In our model, the equilibrium abundance of a species is a function of two factors: the hawk-dove game parameters *V*_*k*_ and the initial species distribution (see the electronic supplementary material, appendix S1.5). If we consider independent ecosystems (no mutual species exchange via immigration), then we may assume that the initial species abundance distributions there are statistically independent from one another. On the other hand, if the two ecosystems are broadly physically similar [13,15–18], then the hawk-dove game parameters may be similar even for isolated systems (examples would include American and European taiga, Ecuadorian and Peruvian rainforest or isolated Mediterranean hyper-saline vents).

We wished to quantify the degree to which similar but independent ecosystems could produce correlated species abundance distributions. For this purpose, we used a paired runs protocol. Each paired run was composed of two simulations. For each simulation, we independently sampled the initial species distribution from the uniform random distribution, while both simulations within a run shared the same *V*_*k*_ values (after each run the *V*_*k*_ were re-sampled uniformly at random in the range from 1 to 1.9 for the subsequent run). After that, we numerically integrated the system to find the equilibrium solution for both simulations within a run. Then, we calculated the correlation between the logarithms of the species probabilities across the two simulations (see example in figure 2*a*). We repeated this procedure 100 times to derive and estimated value of the species abundance correlations.

## 3. Results

**Figure 1.**
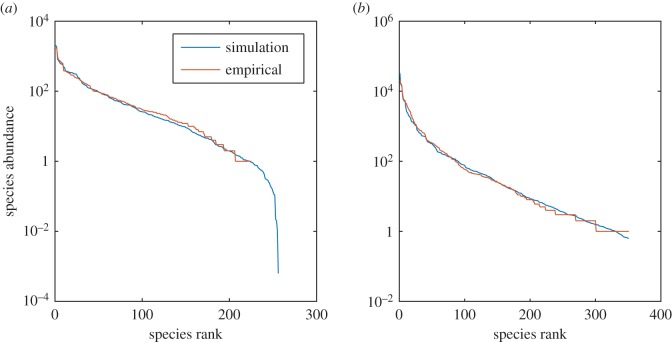
Species abundance distributions (SADs). SADs for two datasets—the Panamanian trees dataset [13] (*a*) and the North-West Mediterranean phytoplankton [14] (*b*). Red, empirical distributions; blue, model results at equilibrium. Simulation parameters for BCI dataset as given in the main text. For the phytoplankton, we used *N* = 9 (needed because we have more species) and *V* was sampled uniformly at random between 1 and 1.99 to generate greater variance. Furthermore, a cut-off at 350 species was used when presenting the histogram to better facilitate comparison with data.

We tested whether our model reproduces the species abundance distribution in the Barro Colorado tree community (BCI)—a standard large-scale dataset for community ecology [13]. As the community contains around 200 species, we chose a value of *N* = 8 (the minimal value capable of producing 200 species in our model) and we sampled each *V*_*i*_ independently and uniformly at random in the range from 1 to 1.9. The starting probability distributions of all 2^*N*^ species were chosen from a uniform random distribution and normalized to sum to one. We simulated the replicator equation (see the electronic supplementary material, appendix S1.3 for details on the simulation protocol) in various random starts and observed the equilibrium state distributions of strategy probabilities frequently converging to the lognormal distribution, which is very different from our starting state—the uniform probability distribution. The same also held true if we initialized the population from a Gaussian distribution. We found an excellent match between the simulation and empirical data (figure 1*a*). We obtained a similar result for a Mediterranean phytoplankton dataset [14,19] (figure 1*b*). A quantitative comparison of our model, an analytic solution to neutral theory [20,21], the lognormal and the gamma (the Gambin model) distributions [22] indicated similar performance for the four models with neutral theory performing best on the BCI dataset while the hawk-dove game performed best for the plankton dataset (see the electronic supplementary material, appendix S1.4 for details).

A mathematical analysis of our model shows how the lognormal distribution emerges to capture the species abundance distribution. As we show in the electronic supplementary material, appendix S1.5, the equilibrium probability distribution for each macro strategy in each simulation is proportional to the products of the equilibrium probabilities of its component micro strategies. For example, the probability of observing the macro strategy *j* = *H*_1_*H*_2_*D*_3_*H*_4_*D*_5_ is proportional to *π*_*j*_ ∝ *p*_*H*_1__*p*_*H*_2__*p*_*D*_3__*p*_*H*_4__*p*_*D*_5__. As all micro strategies are equally represented in the list of all macro strategies, we can view the full collection of macro probabilities as arising from a set of random products over the component micro strategy probabilities. Just like random sums tend to the normal distribution according to the central limit theorem, random products tend to the lognormal distribution, and this explains why the lognormal distribution emerges in our model [23] (the electronic supplementary material, appendix S1.6).

Some species abundance distributions have been described as having a limited degree of multi-modality [24]. In this respect, we note that under certain distributions of *V*_*i*_ values, multi-modal species distributions emerge in our model (the electronic supplementary material, appendix S1.7).

As shown in Methods, our model implies that the equilibrium solution for the population abundances will lie somewhere along a neutral hyperplane. The existence of a whole plane of equilibrium solutions means that, depending on the initial conditions, we may end up with very different equilibrium abundance values for each species across independent runs of the model. At the same time, our formula for the final abundance distribution (see the electronic supplementary material, appendix S1.5) also indicates that the parameters of the hawk-dove games influence the expected abundances of the strategies. This opens up the possibility of finding correlated species abundance distributions across independent ecosystems that have similar values for the hawk-dove game pay-offs.

We used the procedure of paired runs described in the Methods to quantify the expected average correlation between the species abundance distributions across two independent (non-interacting) ecosystems. In 100 simulations, we consistently found a strong average correlation between the log species probabilities of independent ecosystem pairs with a mean correlation value of 0.75 and a standard deviation of 0.08 (*N* = 100, 95% confidence intervals 0.734 and 0.765 for the mean). The simulations led us to conclude that uncoupled ecosystems could indeed show correlated abundance distributions under our model. This value is close to the correlation value of 0.83 ± 0.03 reported for the abundances of the 254 tree species which occur in both Ecuadorian and Peruvian forests [15] (the two sites are located 1400 km apart), since approximately 20% of our paired runs produce a correlation value of 0.83 or greater.

Ecosystems are often subject to unpredictable stimuli which could negatively impact population stability and diversity. Hence, we examined how our neutral equilibrium solutions will react to perturbations. We reasoned that unlike the fully neutral model, our model should show partial recovery from perturbations. To see why, let us assume that we reduce the total number of one strategy type, which we exemplify using the *H*_1_*D*_2_*D*_3_ type. Then, we have created a deficiency in the population of *H*_1_, *D*_2_ and *D*_3_ component strategies. In the one-dimensional hawk-dove game, if the frequency of hawks exceeds the equilibrium frequency of hawks, then the average fitness of doves exceeds the average fitness of hawks. The opposite is true if the frequency of hawks is below the equilibrium hawk frequency [9]. Owing to the assumption of additive fitness, the same conclusions hold in the component games of the multi-dimensional hawk-dove game. Hence, by depleting the population of the *H*_1_, *D*_2_ and *D*_3_ strategies, those micro strategies now have greater fitness than the *D*_1_, *H*_2_ and *H*_3_ strategies (we call this a fitness excess).

After the perturbation, the *H*_1_*D*_2_*D*_3_ is the only macro strategy which experiences a fitness excess across all three of its component micro strategies. Many other species in the population might transiently experience increased fitness (for example the species *H*_1_*D*_2_*H*_3_ probably experiences a fitness excess due to its first two components), but *H*_1_*D*_2_*D*_3_ is the only species which experiences increased fitness along all its three components and therefore has the largest fitness advantage. Therefore, *H*_1_*D*_2_*D*_3_ will experience the greatest growth rate out of all present species and this temporary fitness boost will promote partial recovery of the *H*_1_*D*_2_*D*_3_ from perturbations.

**Figure 2.**
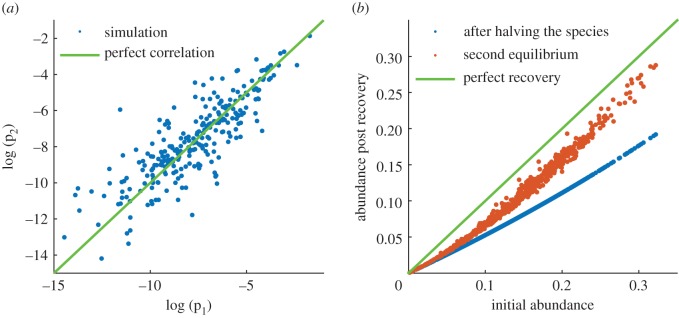
Repeated simulations and perturbations. (*a*) A scatter plot of the log probabilities of species abundances across two independent runs of the model. (*b*) Simulation of how the population reacts to perturbations after reaching an equilibrium. The blue points plot the prevalence of a species after its numbers were halved against its initial prevalence. The red points show population recovery after a new equilibrium has been reached. Green line shows hypothetical perfect recovery.

We simulated a perturbation scenario to examine the degree to which populations will recover from perturbations (figure 2*b*). Following the experimental study from [25], we removed half of the members of one species from a community at equilibrium and subsequently observed its recovery. We plot the results of 600 simulations. In each simulation, we first let the population converge to an equilibrium, then we removed half the members of one species, and we let the population recover. We found there is always recovery with respect to the perturbation (red points are above the blue points, although the recovery is not perfect (red points versus green line). Finally, we observe in our simulations that the more prevalent a species is initially, the better its levels recover. This result qualitatively mirrors what is observed in plant community dynamics [25].

Negative density dependence during a perturbation is not the only criterion which is used to experimentally distinguish coexistence from co-occurrence (co-occurrence can be driven by other processes besides coexistence, like migration from surrounding areas). Another stronger test which is sometimes used is the invasibility test [10], in which a species A is removed from a community, the community is allowed to converge to a new equilibrium, and species A is thereafter reintroduced to a community at a low level. If the species A subsequently increases in prevalence it passes the invasibility test.

In simulations of our model, we find that species who are able to coexist do not pass the invasibility test. In 600 experimental simulations, we reintroduced species A into a community of seven other individuals whose dynamics had previously converged to equilibrium. In all 600 simulations, species A declined in prevalence after it was reintroduced to the community, even though it was introduced to the community at a level which was far below its typical prevalence (mean prevalence without interference 0.13 ± 0.07,  *N* = 600, prevalence enforced at reintroduction 0.01). Therefore, our model illustrates that the invasibility test may be sufficient but is not a necessary criterion by which one can experimentally distinguish coexistence from co-occurrence (i.e. passing the invasibility test guarantees coexistence but failing the test does not rule out the possibility of coexistence).

The reason why the species in our model behave in different ways during perturbation and an invasibility experiment lies in the fact that during an invasibility experiment, the community is allowed to converge to a new equilibrium after A is removed. Thus, before A is reintroduced, the population will contain just the right mix of the different hawk strategies and the reintroduction of A will create an excess of the strategies which the species A embodies. Therefore, A typically will experience increased competition compared to other species and it will decline in numbers after it is reintroduced.

Some recently proposed coexistence models lack robustness against certain deviations in species fitness parameters [5], so we studied whether our model is robust in this respect. In particular, we considered what happens when the additive assumption of pay-offs is violated to some small degree. When our model pay-off matrix is perturbed by a random matrix, we find that biodiversity slowly collapses over time (electronic supplementary material, figure S1). However, this collapse can be easily halted when we add a small self-inhibition term to the model (see the electronic supplementary material, figure S1 and appendix S1.8). Biologically, the self-inhibition term may represent a mechanism such as species-specific pathogen transmission or it could capture the emergence of a species-specific predator. We conclude that our coexistence model is robust to small imperfections which will inevitably occur in real biological systems.

## 4. Discussion

We have proposed a model of species interactions which reveals a new mechanism of coexistence that explains how exponentially more than *N* species can persist on *N* resources. Our coexistence mechanism also holds the potential to explain how models which are niche based at the level of assumptions can lead to outcomes which are consistent with predictions from the neutral theory of biodiversity [26]. In the following discussion, we will highlight the key similarities and differences between our approach and the unified neutral theory (UNT) and then discuss more general implications of our model in the context of coexistence research.

The UNT [4] is both based on the neutrality assumption and produces neutrally stable outcomes. The neutrality assumption is often referred to as the assumption of ecological equivalence and it states that under certain conditions all species have equal fitness. Its introduction was motivated by the success of a similar assumption in neutral theories of molecular evolution, which assumed that all mutations were fitness neutral [27]. Likewise, speciation events are assumed to be fitness neutral in the UNT as well [4,28].

Our theory explains mechanistically how neutral speciation could arise in nature. To see why, consider a two-resource ecosystem composed of the coexisting *H*_1_*D*_2_, the *D*_1_*D*_2_ and the *H*_1_*H*_2_ species. If such a community is in equilibrium, then the strategies *H*_1_ and *D*_1_ produce an equal pay-off in competition for the first resource and the strategies *H*_2_ and *D*_2_ must also produce an equal pay-off in competition for the second resource. Therefore, the *D*_1_*H*_2_ species would have the same fitness as all the other extant strategies and thus such a speciation event would be neutral with respect to the existing population.

Our theory also parallels the neutral theory because both models produce neutrally stable population dynamics. In the UNT, all possible configurations of species are neutrally stable. Neutral stability means that in the absence of stochastic effects, any extant configuration would persist indefinitely while no configuration is self-restoring (perturbations are allowed to accumulate instead of being resisted). Owing to stochastic birth–death and speciation processes, the community dynamics undergoes drift and the system passes through many equally stable population configurations [29]. Our model also leads to a plurality of possible equilibrium configurations, all of which are constrained to lie on a hyperplane which is neutrally stable.

In addition to the above similarities, our model contains a number of features which make it different from the neutral theory of biodiversity and perhaps more closely suited to modelling realistic ecosystems. First, species in our model are not ecologically equivalent. As we showed in the main text, species which are more prevalent in one simulation of our model are likely to be more prevalent in another independent simulation as well and this matches what we find in experiments on well-separated plots in Amazonian forests [15]. The UNT model, by contrast has not been able to explain such long-distance correlations although it has been highly successful in explaining correlations on a smaller scale [13]. Remarkably, this qualitative finding of similarity between independent systems [16,17] holds true whether we are considering the American and Eurasian taigas [18,30], deep hypersaline anoxic basins of the Eastern Mediterranean [31] or Amazonian forests [15].

Another attractive feature of our model as compared with the UNT concerns reactions to perturbations. Species removal experiments and perturbation experiments indicate that many ecosystems react to perturbations in a way which tends to suppress the effects of perturbations [25,26,32–35]. The UNT model again lacks this feature while our model resists most random perturbations (figure 2*b*). Other models have been proposed which are composed of groups of niches within which species show neutral dynamics [36,37]. Such models are also expected to exhibit partial recovery from perturbations. However, in these models the restoring force is expected to be uniformly distributed across all the species within the perturbed niche, while our model shows that it is the perturbed species which experiences the strongest restorative force (see figure 2*b*, and the associated argument in the Results section).

The last two characteristics (resistance to perturbations and a correlation between species traits and species abundance) would be traditionally regarded as characteristics of niche models [10,25]. Thus our model simultaneously shows both niche-like and neutral patterns and could be said to help unify the niche and neutral perspectives on ecosystem dynamics.

In contrast with other attempts to combine niche and neutral theories, we do not need to model factors like ongoing speciation events, stochastic inter-generational dynamics, immigration from a surrounding community or dispersal limitations [38–42]. Even in a fully mixed population with deterministic dynamics and no source of new species, we obtain an approximately lognormal species abundance distribution at equilibrium.

Even though our model shares many similarities with the neutral theory, the lognormal distribution is generated in our model via a different mechanism. The lognormal emerges in our model through repeated application of many multiplicative terms (see the electronic supplementary material, appendix S1.6). This theoretical rationale might help to alleviate some of the criticisms of the lognormal distribution as an ad hoc construct and an improper null model in the context of population ecology [43,44]. Owing to the attracting nature of the hyperplane, the equilibrium is also reached very fast, which may be an advantage over other models where kinetic equilibriums can be slow to emerge and are transient in nature [45].

The central object which enabled us to achieve neutral outcomes through niche mechanisms was the hyperplane attractor structure of our model. We anticipate that even if the details of our model prove to be inaccurate in some respects, this underlying mathematical structure may nevertheless be present in many other models which display neutral and niche dynamics simultaneously. This is likely to be the case because the only way that neutral drift and restorative dynamics can simultaneously coexist is if dynamics are neutral along some directions but restoring on others—the very property which defines an attractor manifold.

In fact, one model with both niche and neutral dynamics is the chemostat model of Posfai *et al.* [5] and here too an attractive hyperplane emerges as a result of competitive dynamics. Although not pure hyperplane models, the emergent neutrality model [46] and the competitive life-history trade-off model of coexistence [47] also lead to multiple regions of trait space where dynamics are approximately neutral within a niche. Hyperplane models have previously proved useful in analysing other complex networks such as neural dynamics [48]. We hope that our model will stimulate further research into attractor hyperplanes in theoretical ecology.

Although our model was structured around the hawk-dove game, this is not a necessary condition for the hyperplane to emerge. The model could be extended to other types of games as the components from which the multi-trait strategies are built. As long as the component games have evolutionarily stable mixed strategies as their equilibriums, our mathematical arguments about the existence of the hyperplane hold.

Our model also offers a new potential solution to the paradox of the plankton. The paradox of the plankton refers to an observation made by Hutchinson that oceanic waters contain fewer than 10 distinct growth resources (light, nitrogen, carbon, iron, phosphorous, silicon and a few potential others) yet it supports more than a hundred stably coexisting species [3]. Hutchinson remarked that this empirical finding contradicted the competitive exclusion principle, which stated that the number of coexisting species in equilibrium should not exceed the number of resources [32]. The contradiction became known as the paradox of the plankton and it has spawned many decades of research and dozens of proposals explaining how the paradox might be overcome [49].

Our model proposes that the number of coexisting species grows exponentially with the number of available resources and the presence of seven to 10 different types of resources is enough to support the coexistence of between 100 and 1000 species: a range which appears to be a good match with what is experimentally observed in plankton communities [3,14]. Thus, our model offers a new potential explanation for the paradox of the plankton and its predictions could be tested further in future empirical work.

One other noteworthy feature of our model is its property that species in our model will fail the invasibility test [10] even though coexistence in the model is clearly stable. In this regard, our model parallels other complex multi-species coexistence models which are also known to fail that test [50]. We hope that a more extended examination of such complex coexistence models will stimulate experimentalists to develop improved tests of coexistence more suitable for complex ecological communities.

Finally, it should be noted that like many other ecological models, our model makes certain simplifications whose effects need to be more comprehensively examined in future models. Our species currently lack inter-individual variability [51] and we have not yet provided a way to incorporate evolutionary processes into the model [52]. Also, the assumptions of approximately additive pay-offs may not completely hold for some species orders where trait trade-offs impose constraints on the pay-off matrix [53]. As we showed in the electronic supplementary simulations, our model displays some robustness to violations of the additive pay-off assumption but the precise limits of robustness and the effects of pay-off correlations need to be mapped more extensively in follow-up research. In the future, it will also be interesting to incorporate considerations of stochasticity and dispersal limitations into our baseline model to facilitate further comparisons between existing models in the literature [29].

## Supplementary Material

Supplementary

## Data accessibility

Matlab code and data to reproduce the figures is available for download from the Dryad Digital Repository: http://dx.doi.org/10.5061/dryad.b06f3s2 [54].
