# Supplementary material for: Species diversity rises exponentially with the number of available resources in a multi-trait competition model
Source: Proc Biol Sci. 2018 Aug 29;285(1885):20181273. doi: 10.1098/rspb.2018.1273 (PMC6125918; doi:10.1098/rspb.2018.1273)
Supplement: Supplementary [file rspb20181273supp1.pdf]

Supplement: species diversity rises exponentially with the number of available resources in a multi-trait competition model

Andres Laan[1,2] and Gonzalo G. de Polavieja[1,2]

[1]Champalimaud Research, Champalimaud Center for the Unknown - Lisbon, Portugal
[2]andres.laan@neuro.fchampalimaud.org, gonzalo.polavieja@neuro.fchampalimaud.org

July 28, 2018

Proceedings of the Royal Society B, doi:10.1098/rspb.2018.1273

1 Appendix

Matlab code and data to reproduce the figures is available for download from Dryad under doi:10.5061/dryad.b06f3s2

1.1 Overview of the hawk dove game

The hawk dove game originates from the field of evolutionary game theory [1]. It was originally meant to model aggressive interactions, but it has subsequently found application in modeling a number of other behaviors such as leadership and co-operation [2, 3]. We present the model in the context of aggressive interactions with the added understanding that the same equations can be used to describe a variety of other scenarios.

The hawk dove game considers two different kinds of agents called hawks and doves, who are identical in all respects except their approach to social interactions. The hawks and doves compete over resource patches which have an average added fitness value of V. The hawks and doves differ in their approach to competition. When two doves meet, they split the resource and each leaves with a pay-off of $\frac{V}{2}$. When two hawks meet, they engage in a violent contest over the resource, which leads the net fitness of the pair to be reduced by C. It is also assumed that the average cost of the contest exceeds the value of the resource ($C > V$). Therefore, a hawk-hawk meeting leads to an average individual pay-off of $\frac{V-C}{2}$. Finally, when a hawk meets a dove, the aggressive tactics of the hawk scare away the dove and the hawk has a pay-off of V while the dove has a pay-off of 0.

The population dynamics in the hawk dove game are described in terms of the frequency of hawks p_H and the frequency of doves p_D with $p_H + p_D = 1$. As will be shown below, this formulation still allows us to study co-existence while it removes the need to specify factors irrelevant to co-existence such as resource dynamics, baseline effects and any lack of time-invariance of those two effects. The evolution of the two mutants is determined by their fitness

functions. The average pay-off of the hawk strategy is $R_H = p_H \frac{V-C}{2} + p_D V$ while the dove pay-off is $R_D = p_D \frac{V}{2}$. The pay-off for the average individual can be written as $R_P = p_H R_H + p_D R_D$. Given these terms, we can write down the replicator equation to specify the time evolution of the two mutants as [4]:

$$\begin{aligned} \frac{dp_H}{dt} &= p_H(R_H - R_P) \\ \frac{dp_D}{dt} &= p_D(R_D - R_P) \end{aligned} \tag{1}$$

Two facts are apparent from the replicator equation. First, the time evolution of each strategy depends on the difference between the pay-off of that strategy and the average population strategy. Hence, any process which equally affects the baseline fitness of all agents does not have an influence on the evolution of the population frequencies of the agents. This is important because it allows the agents to derive the bulk of their fitness from other individual-level processes (i.e. non-social foraging, mating, child rearing) while at the same time allowing us to describe the time evolution of frequencies only in terms of the competitive interactions.

Second, the mathematical form of the replicator equation explains why resource dynamics and population densities do not have an effect on the equilibrium state and its stability. Population density and resource density obviously interact in an ecological context. Higher population density leads to a greater number of social interactions and an increase in the rate of resource consumption. Increased rate of resource consumption in turn leads to decreasing resource levels and a lower average fitness. However, it should be noted that both of these processes influence the hawks and the doves equally and multiplicatively. That is, the overall rate of social competitive interactions $\alpha(N, \rho_V, t)$ is some function of population density N and resource abundance ρ_V and time t and this interaction rate transforms the fitnesses of the agents to $R_{H,t} = \alpha(N, \rho_V, t) R_H$ and $R_{D,t} = \alpha(N, \rho_V) R_D$. The transformed replicator equation is:

$$\begin{aligned} \frac{dp_H}{dt} &= \alpha(N, \rho_V, t) p_H(R_H - R_P) \\ \frac{dp_D}{dt} &= \alpha(N, \rho_V, t) p_D(R_D - R_P) \end{aligned} \tag{2}$$

It has been shown that one can always find a monotonic time-dependent velocity transformation such that any solution to the original replicator equation is also a solution to the transformed replicator equation after the velocity transformation [5]. Therefore, overall resource and population dynamics only change how rapidly population dynamics occur but they have no influence on long term co-existence dynamics. Later in the paper, we define the multi-dimensional hawk dove game, which considers more than one type of resource. The fact that resource density transforms only transient dynamics and not long-term stability also means that we do not need to worry about the relative weighting of the different resources on fitness (see section on Robustness for further details).

As mentioned previously, the hawk dove game is typically used to describe intra-specific competition, while we propose to use it in the study of inter-specific competition. Speaking specifically of aggression, there is some empirical evidence in the literature that this choice may be justified. Studies of Ohio salamanders have described two species, P.cinereus and P.electromorphus [6], with P.cinereus showing both greater intra-specific aggressiveness and also a tendency to dominate inter-specific encounters. This finding certainly provides a qualitative match to an inter-specific hawk dove game structure.

More broadly, several other aspects of inter-specific competition appear to show characteristics consistent with the hawk dove game structure. As one example, we can consider competition for light. Isolated tall plants are able to capture a greater fraction of incoming light and they thus out-compete a group of smaller plants. On the other hand, small plants can also invade a dense group of tall plants, perhaps because crowding now reduces the competitive advantage of tall plants while they also suffer from increased investment into trunk materials- a cost that is absent for the smaller plants. The example of the plants and other qualitative descriptions of inter-specific competition [7] suggest that the hawk dove game might be more broadly applicable as a crude model of competition.

1.2 Determining the equilibrium

According to the replicator equation, the change in the population frequency of all agents of type i (p_i) is given by $\frac{dp_i}{dt} = p_i(R_i - R_P)$, where R_P is the average fitness of a randomly chosen agent and R_i is the average fitness of all agents of type i. At equilibrium, the rate of change must be zero for all types. This is only possible if $R_i = R_j = R_P$ for all co-existing agents (those are all agents j whose $p_j > 0$). Hence, at equilibrium all co-existing agents must have equal average fitness.

We can now apply this principle to determine the equilibrium population state for the hawk dove game. At equilibrium, we must have $R_H = R_D$, leading to $p_H\frac{V-C}{2} + p_D V = p_D\frac{V}{2}$. It can be verified by substitution that $p_H = \frac{V}{C}, p_D = 1 - p_H$ solves the stated equation.

To apply the same idea to the multi-dimensional hawk dove game, we can consider two strategies, $S_{-i,1}H_i$ and $S_{-i,2}D_i$, with $S_{-i,1} = S_{-i,2}$, where S_{-i} specifies the strategies adopted in all other games except i. The condition $S_{-i,1} = S_{-i,2}$ specifies that we are considering two species which share the same strategies for all micro games except micro game i where animal 1 plays hawk and animal 2 plays dove. Next, we compute the pay-off difference $R_{S_{-i,1}H_i} - R_{S_{-i,2}D_i}$. Due to the additive pay-off assumption, this difference is written as $p_{H_i}\frac{V_i-C_i}{2} + p_{D_i}V_i - p_{D_i}\frac{V_i}{2}$, where p_{H_i} is the probability of encountering the hawk strategy in competition for the i-th type of resource against a randomly selected opponent in the population. As before, the equilibrium condition requires the pay-off difference to be zero and thus $p_{H_i} = \frac{V_i}{C_i}$ as stated in the main text based on a more informal argument. Notice that a change in the values of C_i and V_i to $\alpha_i C_i$ and $\alpha_i V_i$ would not change the value of the equilibrium frequency of p_{H_k} and thus the conclusions regarding equilibrium frequencies are robust even if we arbitrarily increased the fitness contributions of some micro games over others.

1.3 Numerical simulation

We define the pay-off matrix A, where element A_{ij} denotes the pay-off of macro strategy i (S_i) against macro strategy j (S_j). Denoting vector π as the vector of macro strategy probabilities (population frequencies), we get that the expected pay-off of macro strategy i (R_i) is equal to the i-th element of the vector $A\pi$ or $R_i = (A\pi)_i$. The pay-off of the average member in the population is $R_P = \pi^T A\pi$. The macro strategy probabilities evolve according to the replicator equation which is written as:

$$\frac{d\pi_i}{dt} = \pi_i(R_i - R_P) \tag{3}$$

In the main text, we defined the pay-offs as arising from an additive structure where the total pay-off of macro strategy S_i is the sum of its pay-offs in each of the N micro games. Therefore, $A_{ij} = \sum_{k=1}^{N} R(S_{ik}, S_{jk}, V_k, C_k)$, where S_{ik} is hawk (H) if strategy i involves playing hawk in micro game k and is dove (D) if strategy i involves playing dove in micro game k. The micro game pay-off function $R(s_1, s_2, V, C)$ is represented by the table:

	H	D
H	$\frac{V-C}{2}$	V
D	0	$\frac{V}{2}$

where the row label gives the micro strategy of player 1, the column label gives the micro strategy of player 2, and the elements of the table mark the resulting micro game pay-off of player 1.

At the individual level, our model is consistent with a variety of different interpretations. All component micro games could occur simultaneously during each interaction or the micro game which occurs could instead be sampled randomly during each interaction between a pair of individuals. Since both assumptions lead to the same replicator equation, we are not constrained to provide a single interpretation of the underlying agent level interaction rules.

1.4 Quantitative comparison of fits

In order to compare the quality of fits, we plotted the cumulative probability distribution of the empirical dataset, the lognormal model and our simulated model on a logarithmic axis (see Figure S5 for illustration). The we calculated the mean absolute deviation between the fits and the empirical distribution and we integrated this absolute error over the whole axis to find the mean absolute deviation between the data and the fit. For the case of simulations, we report the mean error for 20 simulations. The distributions of the simulated distributions were truncated below to allow them to match the empirically reported number of species. For the BCI dataset, report mean absolute errors of 1.6, 4.0, 2.3 ± 0.1 and 1.0 (lognormal, gamma, our model, Hubbell) and for the plankton dataset the corresponding numbers are 4.2, 3.7, 2.9 ± 0.2 and 9.7 (mean $\pm$ standard error in the mean, $N = 20$ simulations). Thus neutral theory outperforms our model slightly for the BCI data while the converse is true for the plankton dataset. To calculate the parameters for neutral theory, we used the methodology and code published in [8].

1.5 Approximate analytical solution

Here, we show that an approximate solution to our model can be expressed in terms of the solution to the individual component hawk dove game models. We define the following notation. $\pi_i(t)$ is the probability of observing macro strategy i at time t. $p_{H_k}(t)$ is the the probability of encountering a hawk player in game k at time t. As before, $p_{H_k}(t) = \sum_{j \in H_k} \pi_j(t)$, where $j \in H_k$ stands for a strategy j which includes playing hawk in game k as its element. $s_{H_k}(t)$ stands for the function which solves the two-player hawk dove game replicator equation with pay-offs V_k, C_k and where $s_{H_k}(0) = p_{H_k}(0)$. In other words, $s_{H_k}(t)$ would capture the the time evolution of the prevalence of the H_i strategy if all players were only participating in the k-th hawk dove game and other parts of their macro strategy did not contribute to fitness. In order to represent macro strategies, we use a binary notation. Macro strategy j corresponds to a binary number t_j, where the i-th element of t_j ($t_{j,i}$) is equal to 1(0) if it involves playing hawk (dove) in game i. For example, if macro strategy j is $H_1\,D_2\,D_3\,H_4$ then $t_j = (1, 0, 0, 1)$.

We define the pay-off of playing the hawk strategy in game k at time t as $R_{k,H}(t)$ ($R_{k,D}(t)$ for playing dove). Furthermore, $R_{k,H}(t) = \frac{V_k - C_k}{2} p_{H_k}(t) + V(1 - p_{H_k}(t))$ (and the analogous equation for the dove). The time derivative of s_{H_j} is obtained from the standard two-player hawk dove replicator equation and is equal to

$$\frac{ds_{H_i}}{dt} = s_{H_i}(R_H - s_{H_i}R_H - (1 - s_{H_i})R_D) = s_{H_i}(1 - s_{H_i})[R_H - R_D]. \tag{4}$$

Based on the definition of our model, we know that the pay-off of the macro strategy j is given by $R_j(t) = \sum_i t_{j,i}R_{k,H}(t) + (1 - t_{j,i})R_{k,D}(t)$.

With the above notation, we write our guess for solution to the overall replicator equation as

$$\pi_j^g(t) = \frac{\pi_j(0)}{M(j)} \prod_{i=1}^{N} s_{H_i}(t)^{t_{j,i}} (1 - s_{H_i}(t))^{1-t_{j,i}}, \tag{5}$$

with $M(j)$ a normalization factor for strategy j, chosen so that at time $t = 0$, the above equation gives the initial condition value $\pi_j(0)$. This trial solution can be examined by taking a time derivate of Equation (5), giving

$$\frac{d\pi_j^g(t)}{dt} = \pi_j^g(t) \sum_{i=1}^{N} \frac{(-1)^{(1-t_{j,i})}}{s_{H_i}(t)^{t_{j,i}} (1 - s_{H_i}(t))^{1-t_{j,i}}} \frac{ds_{H_i}}{dt}. \tag{6}$$

Now, if we use Equation (4) for the value of the derivate terms $\frac{ds_{H_i}}{dt}$, we obtain

$$\frac{d\pi_j^g(t)}{dt} = \pi_j^g(t) \sum_{i=1}^{N} [t_{j,i}(1 - s_{H_i}(t)) - (1 - t_{j,i}) s_{H_i}(t)][R_{k,H}(t) - R_{k,D}(t)]. \tag{7}$$

On the other hand, the replicator equation states that $\pi_j^g(t)$ must satisfy

$$\frac{d\pi_j^g(t)}{dt} = \pi_j^g(t)[R_j(t) - \sum_{z=1}^{N} p_{H_z}^g(t) R_{z,H}(t) + (1 - p_{H_z}^g(t)) R_{z,D}(t)]. \tag{8}$$

If we expand out the terms for $R_j(t)$ and replace $p_{H_z}^g(t)$ with $s_{H_z}(t)$ (we justify this below), then we can see that Equations (8) and (7) are equivalent and therefore Equation (5) is the solution to our model.

The key to this calculation is the assumption that $p_{H_i}^g(t) = s_{H_i}(t)$. To show why this can approximately be the case, we write: $p_{H_i}^g(t) = \sum_{j \in H_k} \pi_j^g(t)$. Each term $\pi_j^g(t)$ is a product of many terms, namely the $\pi_j(0)$, the $M(j)$ and all the s_{H_i}. Note that since all the variables were initialized independently, we can think of this as a product of independent random variables and we can calculate the expected value of the product by multiplying together the expected values of all terms. When we carry out this procedure, we find exactly that $p_{H_i}(t) = s_{H_i}(t)$ in expectation.

We verified with computational simulations that Equation (5) produces very good approximations to our model. In **Figure S2**, you can see that the simulated equilibrium probabilities of our model match very well the approximate analytical solution.

1.6 Log-normality

The approximate analytical solution provides a key insight into the model. First, note that Equation (5) may be thought of as a product of multiple random variables. While the sum of many random variables tends to a Gaussian, the product of many random variables tends to a log-normal distribution. This helps explain why we observe log-normal distributions in our simulations.

1.7 Multi-modality

As mentioned in the main text, certain SADs may be weakly multi-modal. Using our analytical approximation, we can see that one way in which multi-modality can emerge is if one of the V_i values is much larger than all the others (close to C as opposed to close to $\frac{1}{2}$). Because the

V_i interact multiplicatively, this causes the product probabilities to split into two groups: one group is containing the term $\frac{V_i}{C}$ in it, which is approximately 1, the other group contains the term $1 - \frac{V_i}{C}$, which is close to zero. As you can see in **Figure S3**, multi-modal distributions can thus emerge in the SAD. Biologically, this might correspond to a competitive scenario where one type of resource has very low value for survival.

1.8 Robustness

We studied how co-existence will react to perturbations of our model structure. Let the default pay-off matrix of our game be P. Then we consider a modified pay-off matrix $P_m = P + s\Sigma - aI$, where Σ is a matrix with elements sampled from the independent random normal distribution (this represents the perturbation) and I is the identity matrix (representing the self-inhibition term for each species). When both a and s are zero (our regular model), the population dynamics converges to an equilibrium as described in the main text of the paper (**Figure S1**, top left). When we add a small perturbation with s=0.01, we see that stable co-existence degenerates over time (**Figure S1** top middle for time series, top right for histogram of final probability distributions).

If we now also turn on the self-inhibition term (we set a=0.1), we find that co-existence is once again stabilized (**Figure S1**, bottom panels) and a large fraction of the total species will persist at equilibrium. In general, the number of species co-existing will decrease as s increases, but this decrease can always be counteracted by a corresponding increase in a. The results of our simulations are robust to each species having its own private self-inhibition term value which is slightly different from the mean value a (we explored a $\pm 10\%$ level of random variation).

1.9 Micro game contribution variations

In section 1.1 and section 1.2 of the **Appendix**, we presented mathematical claims that many mathematical properties of the equilibriums in our model remain unchanged even if we allow for rescaling of the rewards such that some micro games contribute more to fitness than others. Here we present further evidence from simulations that reward rescaling only changes the dynamics and not the equilibrium. We define N rescaling coefficients $\alpha_1, \alpha_2, ..., \alpha_N$ whose action is to change $V_k := \alpha_k V_k$ and $C_k := \alpha_k C_k$.

For each simulation, we sample the α values independently and randomly from a uniform distribution between 0.2 and 1.2. The we produce two dynamical systems, both of which share the same initial state and V_k values but they differ in the values of alpha. For the first dynamical system, all the alphas are 1 (pay-offs remain unchanged) whereas for the second dynamical system the αs are sampled randomly as previously defined.

Next we transform the V_k values fur both systems using the alphas and we simulate the dynamics of both systems. We monitor how π_i values change across both systems and we visualize them by plotting the $\log(\pi_i)$ values of both systems against each other at various times. As can be seen from an example plot in **Figure S4**, the two systems diverge initially but later re-converge again to have nearly identical $\log(\pi_i)$ values as both systems approach equilibrium. Thus the pay-off rescaling transformation of the second system primarily influences the dynamics of the system but not the equilibrium configuration as we also concluded from mathematical analysis.

2 Supplementary figures

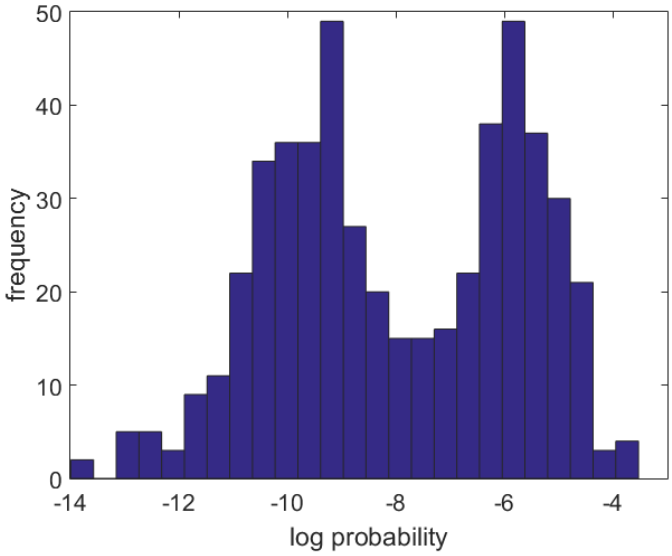

Figure S1: **Example histogram of a simulation which converged to a multi-modal distribution.** For this simulation, $N = 9$, $C = 2$, $V_1 = 1.99$ and V_2 to V_8 was sampled uniformly at random between 1 and 1.4.

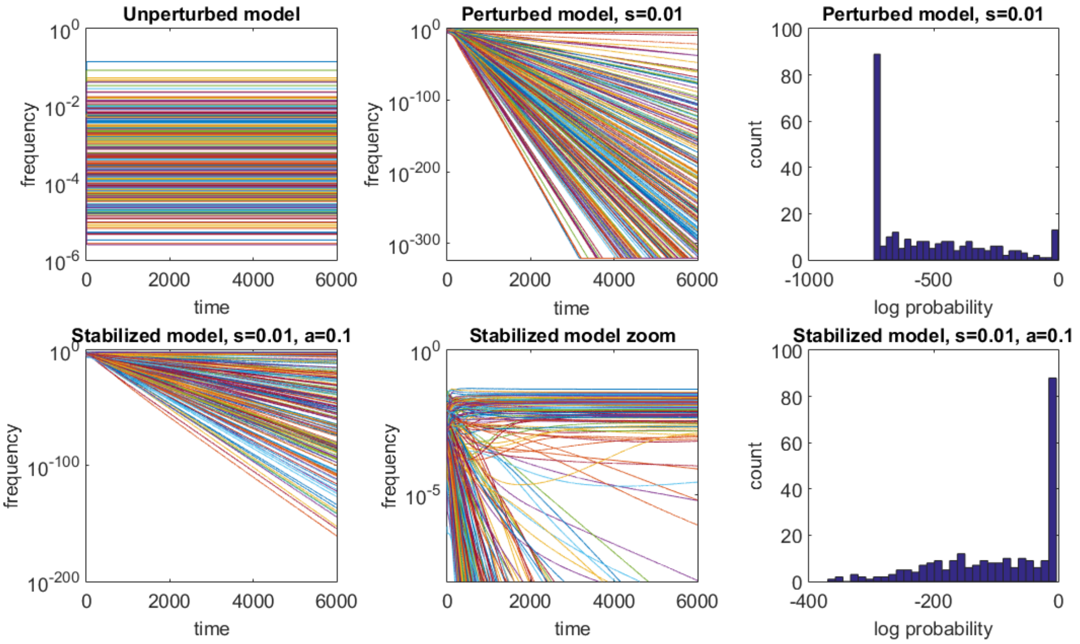

Figure S2: **Study of robustness** Top left: a time series of species probabilities for the unperturbed model $(s, a = 0)$. To middle: same but $s = 0.01$. Top right: a histogram of the log of the probability distributions at the end of the simulation in the top middle figure. Bottom left: a simulation where the self-inhibition term is activated $a = 0.1, s = 0.01$. Bottom middle: a zoom into the bottom left panel area to show the species which stably persist over time once self-inhibition is activated. Bottom right: histogram of log probabilities at the end of the simulation in the left hand figure. Notice the large peak of 85 species near zero which will co-exists in a stable way even in the face of perturbations.

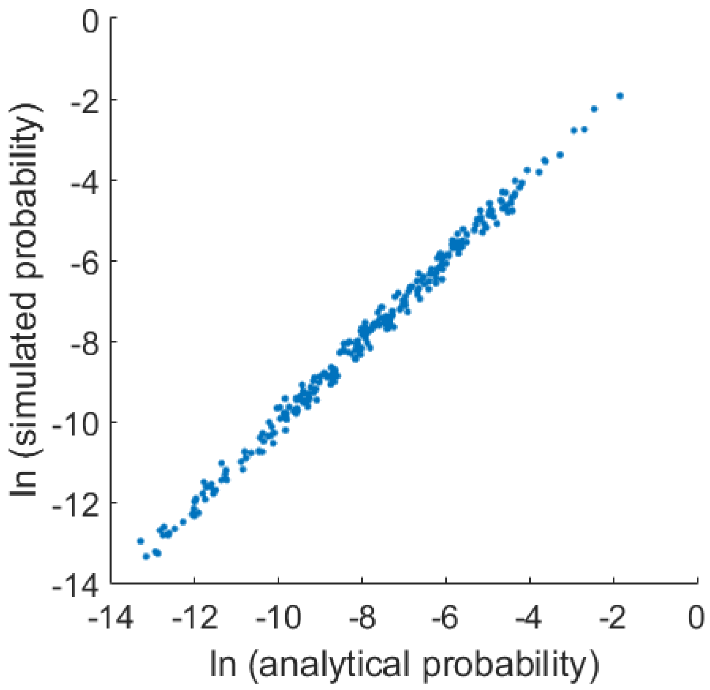

Figure S3: **The analytical formula in Equation (1) gives a good approximation to the simulated results** Probabilities obtained from the approximate analytic Equation (1) and equilibrium solution in simulation. This is a log-log plot to facilitate seeing the entire range of probabilities.

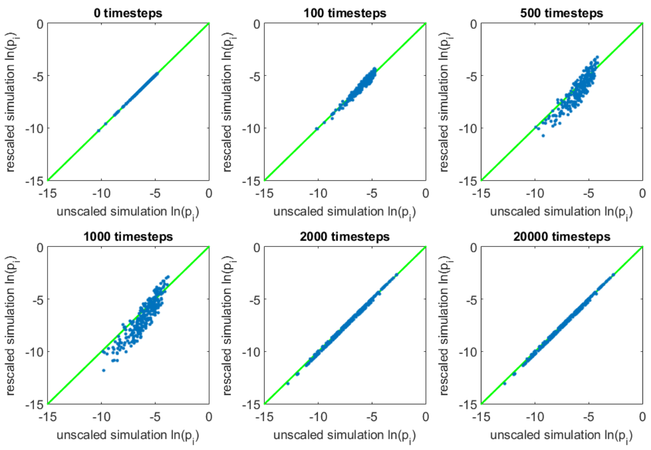

Figure S4: **Comparison of rescaled pay-off dynamics with unscaled dynamics** The x axis marks the log probabilities in the unscaled pay-off model, the y axis gives the log probabilities in the rescaled pay-off model. The simulations proceed from the same initial conditions in parallel and each panel is a snapshot in time (time given in the panel title). Notice how the systems begin in an identical state, diverge and then converge over time. Hence random micro game pay-off rescaling affects the dynamics but not the equilibrium properties.

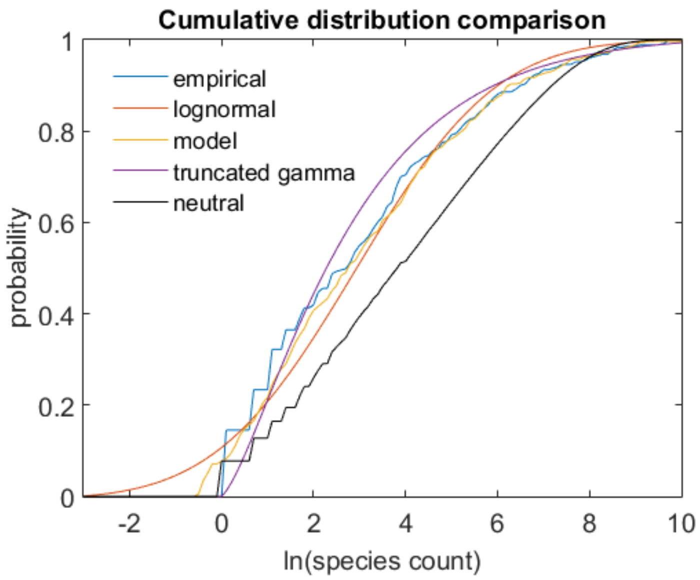

Figure S5: **Comparison of cumulative probability distributions** The Mediterranean plankton dataset is used to illustrate the graphs we used to produce a quantitative ranking of fits.

References

[1] Kokko H, et al. Dyadic contests: modelling flights between two individuals. In: Animal contests. Cambridge University Press; 2013. .

[2] Johnstone RA, Manica A. Evolution of personality differences in leadership. Proceedings of the National Academy of Sciences. 2011;108(20):8373–8378.

[3] Hauert C, Doebeli M. Spatial structure often inhibits the evolution of cooperation in the snowdrift game. Nature. 2004;428(6983):643.

[4] Broom M, Rychtár J. Game-theoretical models in biology. CRC Press; 2013.

[5] Hofbauer J, Sigmund K. Evolutionary games and population dynamics. Cambridge university press; 1998.

[6] Deitloff J, Adams DC, Olechnowski BF, Jaeger RG. Interspecific aggression in Ohio Plethodon: implications for competition. Herpetologica. 2008;64(2):180–188.

[7] Yodzis P. Competition for space and the structure of ecological communities. vol. 25. Springer Science & Business Media; 2013.

[8] McGill BJ, Maurer BA, Weiser MD. Empirical evaluation of neutral theory. Ecology. 2006;87(6):1411–1423.
